# Nanomedicine in maternal viral infections: advancing prenatal therapies for fetal protection

**DOI:** 10.1186/s11671-026-04449-1

**Published:** 2026-02-02

**Authors:** Akmal Zubair, Syeda Maryam Hussain, Ghazala Ambreen, Ranya Mohammed Elmagzoub, Muhammad Muaz Arif, A. Alhadhrami

**Affiliations:** 1 Department of Biotechnology, Quaid-i-Azam University, Islamabad, Pakistan; 2 Institute of Animal Sciences, PIR Mehr Ali Shah Arid Agriculture University, Rawalpindi, Pakistan; 3 Riphah Institute of Pharmaceutical Sciences, Riphah International University, Lahore, Punjab Pakistan; 4 Department of Biology and Biotechnology, Faculty of Science and Technology, Al-Neelain University, Khartoum, Sudan; 5 Department of Chemistry, College of Science, Taif University, P.O. Box 11099, Taif, 21944 Saudi Arabia

**Keywords:** Pregnancy, Fetus, Nanomedicine, Viral infection, Congenital infection, Mother health

## Abstract

Viral infections during pregnancy can lead to several adverse outcomes, including miscarriage, stillbirth, intrauterine growth restriction, and neonatal complications, which may manifest congenital malformations and organ dysfunction. Infants who exhibit symptoms following maternal infection tend to have poorer health outcomes compared to their asymptomatic counterparts. Various viruses are known to cause birth defects, with the most common being cytomegalovirus (CMV), rubella virus, hepatitis B and C viruses, herpes simplex viruses 1 and 2, human herpesvirus 6 (HHV-6), Zika virus, and human immunodeficiency virus. In this review article, we examined the most prevalent maternal viral infections that can cross the placental barrier and affect the fetus, potentially resulting in severe damage. Nanomedicine emerges as a promising candidate capable of traversing the placenta to mitigate viral infections in the fetus, thereby minimizing damage. We explored several classes of nanoparticle-based clinical approaches, along with their associated complications and success rates in various trials targeting different types of maternal viral infections. Additionally, we discussed several nanomedicines that can effectively combat viral infections during pregnancy, serving as potential safeguards for both the mother and the fetus.

## Introduction

Pregnancy may exacerbate the symptoms of several different infectious diseases, making treatment more challenging [1]. Approximately 20,000 infants are born each year in the United States to mothers infected with the hepatitis B virus (HBV) [2]. In the absence of postexposure prophylaxis, approximately 6000 children would develop a persistent HBV infection, leading to the premature death of around 1500 due to chronic liver disease. Furthermore, nearly 4000 newborns would acquire a persistent HCV infection. To combat this issue, perinatal HBV prevention programs involve screening pregnant women for HBV and providing immunizations for their newborns [3]. Although the risk of contracting hepatitis C during pregnancy appears lower than that of hepatitis B, there is still a possibility of hepatitis C transmission during pregnancy. Acquisition of the Hepatitis E virus during pregnancy, especially in the third trimester, carries the potential for severe complications for both the mother and the unborn child. It has been associated with an increased incidence of unexpected abortions, stillbirths, and fulminant hepatitis in both mothers and their newborn offspring, which is a very serious form of the disease [4]. Rubella is a highly infectious childhood disease that can lead to significant epidemics every few years. This illness is preventable through vaccination, and in developed countries, outbreaks primarily occur among unvaccinated individuals. Rubella virus (genus Rubivirus) is an enveloped, positive-sense RNA virus currently classified in the family Matonaviridae, a change from earlier classifications that placed rubella within the Togaviridae family. It causes a generally mild febrile exanthem in immunocompetent children and adults. Natural infection typically induces long-lasting, type-specific humoral immunity; clinically apparent reinfection after documented natural disease is uncommon and, when it occurs, is usually asymptomatic or very mild. The primary maternal–fetal concern is congenital rubella syndrome (CRS). When primary maternal infection occurs early in pregnancy, the virus readily crosses the placenta, infects the fetus, and disrupts organogenesis. The likelihood of fetal infection and teratogenesis is highest during the first trimester. Maternal infection between weeks 0 and 11 is associated with fetal infection and congenital defects in approximately 65–90% of cases (classically cited as up to 85–90% in the first 8–10 weeks). The risk decreases to roughly 50% at 13–16 weeks and is substantially lower after 20 weeks, when infection more commonly results in asymptomatic congenital infection rather than the full CRS phenotype. Typical CRS manifestations include sensorineural hearing loss, congenital cataracts, pigmentary retinopathy, patent ductus arteriosus and other cardiac lesions, microcephaly and neurological impairment, and endocrine disorders. Infected infants may shed the virus in respiratory secretions and urine for many months. Population burden and public-health impact are substantial where rubella vaccination is not widely implemented. Modelled global estimates indicate that tens of thousands of infants are born with CRS annually; recent analyses estimate on the order of ~ 32,000 (model median estimate for 2019; 95% CI 13,000–60,000) CRS cases globally, concentrated in countries without routine rubella-containing vaccine (RCV) programmes, and global surveillance/underreporting mean reported case counts are underestimates. The World Health Organization and CDC emphasize that vaccination before pregnancy is the only reliable prevention strategy, and that maternal serologic screening (IgG) is a routine prenatal measure to identify women susceptible to rubella and guide counselling and vaccination postpartum. Rubella virus infections that occur during the first trimester of pregnancy are associated with a considerable teratogenic risk; however, most rubella virus infections that occur after birth are harmless and heal on their own via spontaneous healing. It is estimated that between 80 and 85% of cases are affected by congenital rubella syndrome (CRS), which is a potentially lethal condition. It is possible for a sizeable section of the population that has not been vaccinated, including pregnant women, to remain asymptomatic when they are infected with RV [5–7].

 Cytomegalovirus (CMV) is among the most prevalent viruses that may induce both acute and chronic health issues. CMV is defined by a capsid, an incompletely closed envelope, a genome of 235 kb, and double-stranded linear DNA; it belongs to the herpesvirus family. Immunomodulatory glycoproteins are encapsulated inside an outer membrane. Contracting cytomegalovirus, which often results in a mild and self-limiting illness, may occur in individuals of any age. Seroprevalence among reproductive-aged women varies from 50 to 80%, demonstrating an inverse correlation with socioeconomic level. Intrauterine transmission of primary CMV infection during gestation may result in severe difficulties for the developing fetus, including growth retardation, jaundice, hepatosplenomegaly, and central nervous system abnormalities. Individuals who are otherwise asymptomatic may have potentially irreparable hearing loss or brain damage over time. The virus may still be transmitted during pregnancy, even if the mother has immunity. The likelihood of infection is uniform throughout the first trimester, including the period immediately before conception and the subsequent weeks of gestation [8–10]. After the first exposure, the herpes simplex virus (HSV) causes a dormant infection, which has the potential to become active again. It is a neurotropic virus that belongs to the herpesvirus family and includes two different types. The Herpes Simplex Virus type 1 (HSV-1) and the Herpes Simplex Virus type 2 (HSV-2) [11–13].

 In the 1980s, Herpes simplex virus type 2 (HSV-2) was the predominant cause of genital herpes worldwide. However, epidemiological studies from the 1990s onward have revealed a gradual increase in genital infections caused by Herpes simplex virus type 1 (HSV-1), particularly in high-income countries. Recent global data estimate that approximately 491 million people aged 15–49 years are infected with HSV-2, while about 3.7 billion are infected with HSV-1, indicating widespread potential for both oral and genital transmission [14]. The shift toward HSV-1 genital infections is largely attributed to changing sexual behaviors, including increased oral–genital contact and declining childhood HSV-1 seroprevalence, which leaves young adults susceptible to primary genital HSV-1 infection. From a maternal–fetal perspective, this trend has significant clinical implications. Primary genital HSV infection acquired in the third trimester carries the highest risk (30–50%) of neonatal transmission, as maternal seroconversion and transplacental transfer of protective antibodies have not yet occurred. In contrast, women with recurrent infection or long-standing seropositivity exhibit a much lower transmission rate of less than 1%, owing to preexisting neutralizing antibodies that limit viral shedding during delivery [15]. Neonatal herpes, though rare (occurring in approximately 1 in 3200 to 1 in 10,000 live births globally), is associated with severe outcomes such as disseminated infection, central nervous system involvement, and mortality rates approaching 60% in untreated cases. These risks underscore the need for type-specific HSV-1 and HSV-2 testing among pregnant women who present with genital lesions or have a partner with a history of herpes. Viral infection can be managed with suppressive antiviral therapy (acyclovir or valacyclovir) starting at 36 weeks of gestation, and cesarean section should be arranged in cases of active lesions or prodromal symptoms during labor to reduce vertical transmission. Therefore, the epidemiologic shift from HSV-2 to HSV-1 genital infections necessitates increased obstetric vigilance, comprehensive testing protocols, and early antiviral interventions to prevent adverse neonatal outcomes [16]. The presence of HSV infection offers considerable dangers to both the mother and the newborn baby [17, 18]. A spontaneous abortion may take place in as many as 25% of pregnancies when transplacental transmission takes place before to the twenty-week mark of gestation. There is no evidence to suggest that HSV infections in the first trimester of pregnancy increase the risk of spontaneous abortions. This is even though human papillomavirus (HPV) infections inside the uterus may occur later in the pregnancy. The first episode of genital herpes, regardless of whether it creates symptoms, raises the risk of foetal development and premature delivery. This is true even if the symptoms do not manifest themselves. It is important to note that this risk is modified for recurrent infections that do not manifest any symptoms. The human immunodeficiency virus, often known as HIV, is a retrovirus that causes AIDS by leading to the destruction of helper T cells in the immune system. It takes some time for this to take place, since the number of these cells steadily diminishes over time. The pathogenesis of HIV involves a series of processes that lead to the deterioration of the immune system, primarily due to the loss of CD4 + T lymphocytes [19]. The infection begins with the binding of the viral envelope glycoprotein gp120 to the CD4 receptor on host cells, including T helper cells, macrophages, and dendritic cells. Subsequently, gp120 binds to the co-receptors CCR5 or CXCR4, facilitating fusion of the viral membrane with the host cell membrane and allowing viral entry [20]. The viral RNA is then reverse-transcribed into DNA by reverse transcriptase and integrated into the host genome by integrase, forming a provirus that can remain latent or actively produce new viral particles [9]. HIV replication causes chronic immune activation, persistent inflammation, and gradual depletion of CD4 + T cells through direct viral killing, apoptosis of uninfected bystander cells, and immune-mediated mechanisms. The virus also employs immune evasion strategies, including high mutation rates, glycan shielding, and downregulation of major histocompatibility complex (MHC) molecules by the Nef protein. As CD4 + T cell counts decline, the immune system becomes incapable of combating opportunistic infections and cancers, signaling progression to Acquired Immunodeficiency Syndrome (AIDS) [21, 22]. Without treatment, AIDS is fatal; however, antiretroviral therapy (ART) effectively suppresses viral replication, restores immune function, and transforms HIV into a manageable chronic condition [20, 23, 24]. A mother’s infection can be transmitted to her unborn child through the placenta in cases of intrauterine transmission. A newborn may acquire the virus after childbirth through the mother’s blood or vaginal secretions, known as intrapartum transmission. Additionally, a baby can contract the virus through breastfeeding, which is referred to as postpartum transmission [25, 26].

The placenta is a versatile organ that not only facilitates the exchange of nutrients and gases between the mother and fetus but also plays a vital immunological and physical role by blocking the invasion of pathogens, such as viruses. It has a complex cellular structure composed of syncytiotrophoblasts (SCTs), cytotrophoblasts (CTBs), and Hofbauer (HB) cells, which are important in regulating immune defense and maintaining fetal tolerance. Although the placenta serves as a protective barrier, certain viruses have developed specialized mechanisms to cross or bypass it, leading to congenital infections and adverse pregnancy outcomes [27].

One of the earliest immune cells to encounter invading pathogens are Hofbauer (HB) cells, which are fetal-origin macrophages located in the chorionic villi stroma. These cells exhibit both pro-inflammatory and anti-inflammatory responses depending on the level of infection and placental development. When infected by a virus, HB cells recognize viral components through pattern recognition receptors (PRRs), such as Toll-like receptors (TLRs), and initiate antiviral responses by releasing cytokines and performing phagocytosis. However, some viruses, including Zika virus (ZIKV) and HIV, can exploit these cells as sites for replication, facilitating viral persistence and even vertical transmission [28].

The deepest layer of the placenta, composed of multinucleated syncytiotrophoblasts (SCTs), possesses potent antimicrobial and antiviral properties. Their non-fenestrated, continuous structure and the absence of intercellular junctions restrict pathogen access, rendering SCTs resistant to infection by a wide range of viruses. Additionally, SCTs produce type III interferons, antimicrobial peptides, and exosomes carrying antiviral microRNAs, all of which contribute to the placenta’s antiviral defense against viral invasion. This innate immune response plays a crucial role in preventing the translocation of most viruses across the placenta under normal physiological conditions [29]. Conversely, cytotrophoblasts (CTBs) in the lower part of the syncytiotrophoblast (SCT) layer, which contribute to placental growth, are more vulnerable to viral infiltration. They become susceptible because they express surface receptors that mediate viral binding and internalization [30]. For example, receptors such as TIM-1 (T-cell immunoglobulin and mucin-domain containing-1), AXL, and Tyro3, which belong to the TAM receptor family, have been shown to play a significant role in mediating viral entry, particularly in the case of the Zika virus. ZIKV exploits these receptors to enter trophoblasts and HB cells, disrupting placental integrity and allowing the virus to be released into the fetal compartment [31]. In general, although the placenta serves as an effective barrier against most pathogens, certain viruses have evolved sophisticated mechanisms to parasitize placental cells via receptor-mediated pathways, breaching the maternal-fetal barrier and resulting in congenital infection [32].

### Viral infections during pregnancy

**Fig. 1:**
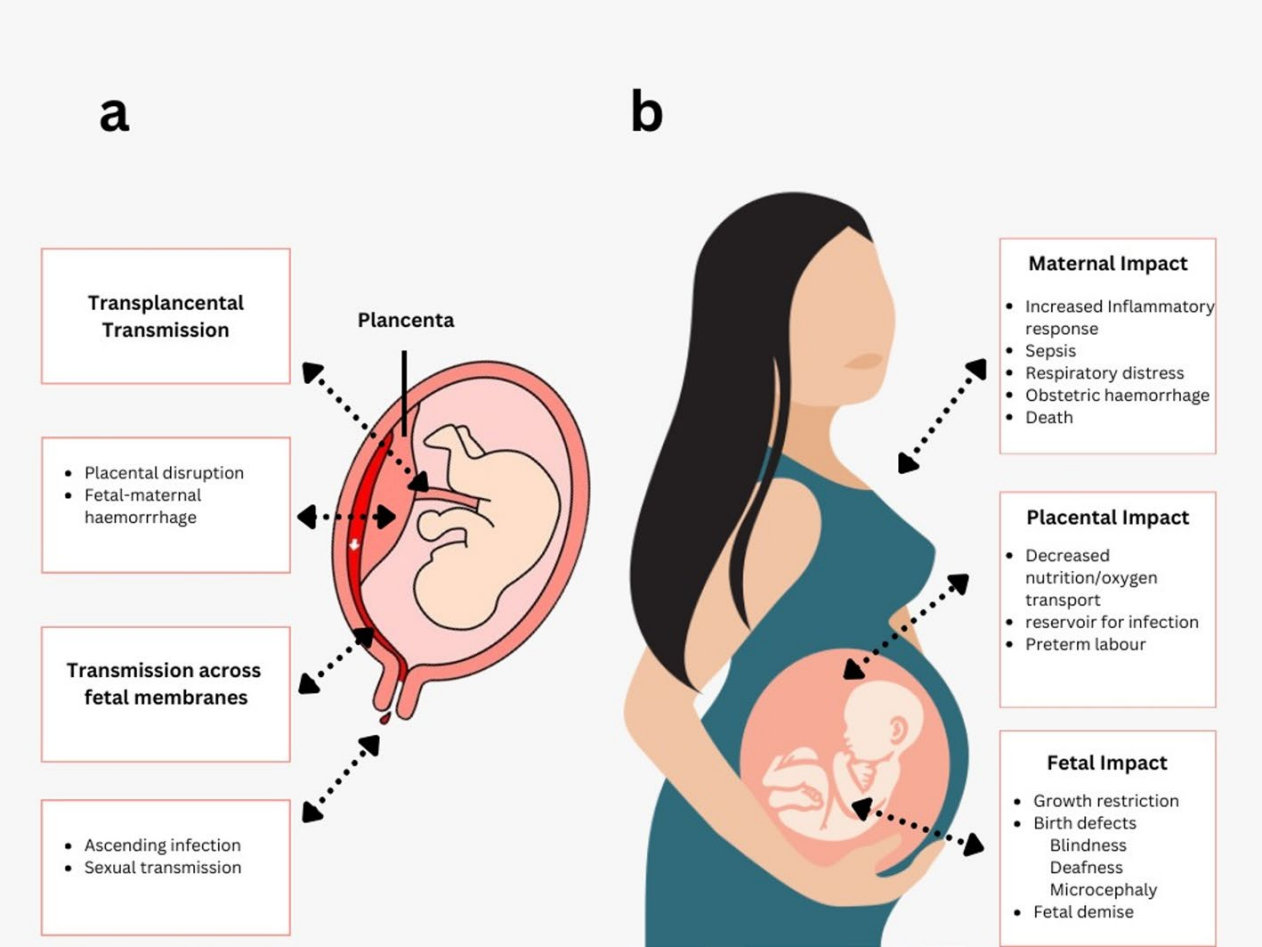
Represents the viral infection that can pass the placental barrier and cause various infections. The placenta barrier provides significant protection to the fetus, and some viruses are still able to cross this barrier and induce significant congenital problems in the fetus

The human microbiome contains a considerable amount of viral DNA, often known as the virome. This part of the virome comprises a diverse mix of endogenous retroviruses, eukaryotic viruses, and bacteriophages [33]. It is gaining growing recognition as a crucial regulator of various bacterial populations and their functions. Even though the great majority of viruses are harmless, certain dangerous viruses can penetrate the maternal-fetal barrier and alter the placenta’s activities, which may result in disease in the fetus. These pathogenic viruses have the potential to cause fetal illness [34]. Table 1; Fig. 1 represent the most prevalent viruses that affect both mother and fetus.

In general, women tend to exhibit stronger immune responses than men. This means their bodies are often more adept at recognizing and combating viruses in the early stages of infection. For instance, in the case of HIV, women typically present with lower viral loads during the initial phases of infection compared to men. However, despite this stronger initial control, women may experience more rapid immune system deterioration over time due to elevated immune activation and inflammation levels [35]. A similar trend is observed with other viruses, such as HBV and HCV, where women generally clear the virus more effectively but are also at an increased risk of heightened immune responses that can lead to liver damage. In the case of HPV, women’s immune systems often clear the virus without any symptoms. However, if the virus persists, it can lead to cervical cancer, particularly if the immune system becomes less effective due to aging or other health factors. In contrast, with SARS-CoV-2, the virus that causes COVID-19, women generally experienced better outcomes than men, including lower rates of hospitalization and death [36, 37]. This is likely attributed to stronger innate and adaptive immune responses in women. Nevertheless, women were more likely to report symptoms of long COVID, which may be associated with that same heightened immune activation. Hormones such as estrogen and progesterone, along with variations in genes located on the X chromosome (of which women have two copies), significantly influence immune responses. However, this enhanced immunity has a drawback: it may result in increased autoimmune symptoms or chronic inflammation, potentially exacerbating long-term health outcomes. While women may initially combat viral infections more effectively, the increased immune activation can occasionally have adverse effects, resulting in distinct health challenges that researchers and healthcare professionals must consider in diagnosis, treatment, and vaccine development [38, 39]. During pregnancy, the maternal immune system undergoes unique adaptations to allow tolerance of the semi-allogeneic fetus while still maintaining the capacity to respond to infections. These changes include a finely balanced shift between pro-inflammatory and anti-inflammatory responses, which vary across different stages of gestation. In early pregnancy, a controlled pro-inflammatory environment supports implantation, whereas later stages are characterized by immune tolerance to protect the fetus. This immunological reprogramming can increase maternal susceptibility to certain viral infections and alter disease progression compared to the non-pregnant state [40].

Several viral infections, such as influenza, Zika virus, cytomegalovirus, and HIV, are known to cause more severe outcomes during pregnancy. This is also mediated by changes in hormones, altered cytokine signaling, and adjustments of both innate and adaptive immune responses. As an example, impaired antiviral response to interferon and impaired natural killer (NK) activities may impede effective clearance of the virus. Moreover, the aspect of maternal viral infections has direct effects on fetal health, including developmental defects and vertical transmissions of the virus, which further indicates the importance of preventive as well as therapeutic interventions [41].

Nanomedicine presents a strong prospect for resolving the issues when it comes to delivering therapeutic agents with an increased therapeutic effect and reduced off-target consequences. NP-based platforms can be developed to enhance drug stability, optimal placental drug delivery where necessary, and specific delivery of antivirals or vaccines to maternal tissue. Notably, nanoparticles have the potential to overcome biological barriers, regulate immune responses, and mediate controlled delivery of a therapeutic agent, and would therefore be ideal for the control of the delicate immunological equilibrium of a foetus [42]. The direction of future research needs to revolve around maximizing the safety, biocompatibility, and targeting specificity of the nanoparticles for maternal-fetal health. In order to reap the benefits of nanoparticles in clinical translation, a more fundamental knowledge of nanoparticle-maternal immune interactions and nanoparticle-placental barrier interactions is required. Given the developments in the three fields of immunology, virology, and nanotechnology, it is possible to devise more effective interventions to reduce the effects of maternal viral infections and safeguard both the mother and child by integrating the field insights [43].

### Viral susceptibility in pregnancy

From an evolutionary perspective, it is essential to limit inflammatory immune responses that could lead to the rejection of the fetus during gestation, while simultaneously enhancing anti-inflammatory responses that promote the transfer of maternal antibodies to the fetus. Such immune reactions, if unchecked, may result in fetal rejection. The significant immunological changes that occur during pregnancy are primarily driven by hormones responsible for these adaptations. Research has shown that levels of estrogens, including estradiol and estriol, as well as progesterone and glucocorticoids, increase during pregnancy. These hormones not only modify the transcriptional signaling of inflammatory immune responses at the maternal-fetal interface but also regulate these responses throughout the body [6, 44]. The pathogenesis of disease during pregnancy is influenced by several factors, including the decreased activity of natural killer cells, inflammatory macrophages, and type 1 helper T cells (Th1), as well as the production of inflammatory cytokines. Additionally, the increased activity of regulatory T cells and the synthesis of anti-inflammatory cytokines also contribute to disease pathogenesis. There is a correlation between the severity of diseases that are alleviated by inflammatory responses, such as influenza and malaria, and the severity of disorders that are exacerbated by inflammatory responses, such as multiple sclerosis, during pregnancy. In other words, the severity of these disorders tends to decrease during pregnancy [45]. The heightened inflammatory responses necessary for managing and eliminating pathogens can negatively impact pregnancy outcomes in the case of various viral infections. These responses are crucial for treating infections and removing pathogens. The interactions between hormones and the immune system are reciprocal, affecting not only pregnancy outcomes but also the extent to which women are susceptible to illness [46].

## Cytomegalovirus

Cytomegalovirus, or CMV as it is more often abbreviated, is a DNA virus that belongs to the Herpesviridae family of viruses. Cytomegalovirus (CMV) stands as the predominant viral infection transmitted vertically from mother to fetus during fetal development, contributing to a broad spectrum of congenital abnormalities [47]. These conditions may cause intellectual disability, hearing loss, and vision loss, in addition to microcephaly, calcifications in the brain, and organ dysfunction. The herpes simplex virus, commonly referred to as HSV, is typically spread from person to person through the exchange of contaminated bodily fluids, which may encompass blood, saliva, urine, breast milk, and various other substances [48]. After a person has been infected with the virus, the virus can persist in the hematopoietic cells of their bone marrow for the remainder of their lives [49]. This may happen even if they have received treatment for the infection. On the other hand, bad pregnancy outcomes are more likely to be caused by a primary infection that arises during pregnancy rather than the reactivation of a disease that has been present for a longer length of time [48]. This is because primary infections are more likely to be caused by a primary infection that develops during pregnancy. The stage of gestation of the mother’s pregnancy at the time of infection influences both the severity of the illness and the repercussions it has for the fetus. This lends credence to the idea that changes in the immunological state of the mother and the interface between the mother and the fetus play a significant role in CMV vertical transmission [49, 50]. Although the precise pathophysiology of CMV has not been fully elucidated, it is known that the severity of the infection and the repercussions for the developing fetus are influenced by the gestational age of the mother when she contracts the virus. Recent research suggests that CMV may infect the placental pericytes before going on to infect the fetus [51]. In addition, pregnant women who are infected with CMV have higher levels of cytokines such as TNF-, IL-1, IL-10, IL-12, IL-15, IL-17, and CXCL10, all of which have the potential to cause a range of pregnancy issues or major health problems to the baby [52]. These elevated levels of cytokines have been linked to a number of adverse birth outcomes. Depending on the circumstances surrounding the pregnancy, these problems might include premature delivery, a low birth weight, and hearing loss either at the time of birth or at a later point in the individual’s life [53]. Cytomegalovirus (CMV) is a leading cause of congenital infections worldwide and can result in severe neonatal complications, including sensorineural hearing loss, neurodevelopmental impairment, and intrauterine growth restriction. Although the pathogenesis is well studied, there are still limited treatment options, especially during pregnancy. The existing antivirals, i.e., ganciclovir and valganciclovir, suppress the replication of the virus, whereas they are not recommended in pregnancy because of their teratogenicity and maternal toxicity. The new antiviral Letervir, which attacks the CMV terminase complex, is promising and may prove valuable during transplantation prophylaxis, with limited evidence of its safety and effectiveness in pregnancy. The lack of a licensed CMV vaccine further complicates CMV elimination and emphasizes the need to use innovative approaches to treatment [54].

The inadequacy of standard antivirals reinforces the possible applicability of antiviral approaches utilising nanomedicine in the management of maternal CMV infections. Nanoparticle-based drug delivery might drive the drug bioavailability and systemic toxicity, as well as allow targeted drug delivery across the maternal? Placenta barrier, thereby benefiting maternal health with a fetal risk reduction. In addition, nanotechnology has possibilities of combination therapy, sustained release formulations, and a probable CMV vaccine development platform, and all these have the potential to break the barriers of CMV management. Therefore, this study of CMV in the maternal-fetal health domain not only highlights the existing problems of treatment but also offers an excellent case to develop nanomedicine solutions as safer and more effective therapeutic alternatives [55].

**Table 1:** Represent different viruses can cross the placenta and damage the fetus

Viruses	Attachment	Genome size	Genome nature	Complications in fetus and pregnancy	References
Hepatitis B	Sodium taurocholate transporting polypeptide (NTCP)	3.2 kb	DNA virus	Placenta abruption, preterm birth, gestational hypertension, and fetal growth restriction	[56]
Hepatitis C	Claudin-1, occludin, SR-B1, LDLr,	9.6 kb	RNA virus	Low birth weight, preterm delivery, and fetal malformations	[57]
Rubella virus	Myelin oligodendrocyte glycoprotein	9.7 kb	RNA virus	Congenital Rubella Syndrome, hearing, vision and intellectual problems	[58]
Immunodeficiency virus	LIF (LIFR)	9.7 kb	RNA	Spontaneous early abortion, low birth weight babies, and stillbirths, preterm labour, preterm rupture of membranes	[59, 60]
Cytomegalovirus	PDGFRα	235 ± 1.9 kb	DNA virus	Blindness, jaundice (yellow skin and eyes), an enlarged liver, an enlarged spleen, low birth weight, small head size, problems with the nervous system	[61]
Herpes simplex virus	HveB and HveC	152 kb	DNA viruses	Eye or skin lesions, meningoencephalitis, disseminated infections, or foetal malformations	[62]
Zika virus	AM (TYRO3, AXL and MER) and TIM (T-cell immunoglobulin and mucin domain	11 kb	RNA virus	Birth defects, including microcephaly and other brain abnormalities.	[63]
COVID-19	ACE2, TMPRSS2 and CD147	29.8 kb	RNA virus	Preterm birth, preeclampsia, and perinatal death	[64]

These are typically the most common representative viruses that were highlighted in this article

### Herpes simplex virus

One of the most widespread infections globally: about 67% of people aged 0–49 carry HSV-1. HSV-1 is commonly acquired in childhood and causes oral infections (“cold sores”), though adults can also acquire genital HSV-1 via sexual contact. HSV-2, typically sexually transmitted, causes genital herpes [65].

In adults, herpes simplex virus (HSV) infections sometimes produce no or minor symptoms. However, a pregnant woman’s susceptibility to viral infections, particularly HSVs, may increase due to alterations in the maternal immune system [66]. This is because the immunological status of the mother shifts from being more inflammatory in the early stages of pregnancy to being less inflammatory in the later stages [67]. The most common way for a newborn to get infected is by vertical transmission when the infant comes into touch with viral sores in the vaginal canal shortly after birth [49]. Although its mode of transmission from mother to kid during pregnancy remains unknown, this has been the case. Consequently, when a pregnant individual contracts herpes simplex virus (HSV) infection, there is an elevated chance of transmitting the virus to the newborn vertically. This, in turn, heightens the risk of herpes simplex encephalitis, chorioretinitis, and cerebral calcification in infants, all of which can result in mortality rates ranging from 50% to 80% if left untreated [68].

### Rubella virus

Global reported cases of rubella have dropped dramatically by 97%, from about 670,894 cases in 2000 to 17,865 cases by 2022. As of January 2024, 175 of 194 countries have integrated the rubella vaccine into their national immunization programs, with estimated vaccine coverage around 69% World Health Organization. Some regions have achieved elimination of rubella; for instance, the Americas eliminated rubella in 2015, and Australia did so in 2018. However, outbreaks continue in regions with lower immunization coverage, notably Africa and Southeast Asia, where congenital rubella syndrome (CRS) remains a concern [69].

The extremely contagious rubella virus belongs to the family Togaviridae. Nonetheless, the rubella virus can trigger necrosis in syncytiotrophoblasts [70], enabling it to breach the placental barrier. This significantly heightens the likelihood of miscarriage or stillbirth for pregnant women who become infected [71]. The virus is airborne and typically only makes healthy adults feel a little under the weather with a low-grade fever. Serious birth abnormalities, including those of the eyes, ears, heart, brain, and speech, may be the consequence of an infection in the womb or shortly after delivery [72].

### Human immunodeficiency virus

By the end of 2024, approximately 40.8 million people were living with HIV globally—39.4 million adults (15+) and 1.4 million children (0–14). New infections in 2024: around 1.3 million (1.0–1.7 million), including 120,000 children; this marks a 40% reduction since 2010 [73]. HIV-related deaths in 2024: approximately 630,000 (490,000–820,000), reduced by 54% since 2010. Regarding care: 87% of people with HIV knew their status; 77% were receiving antiretroviral therapy; and 73% of those had achieved viral suppression [20, 74, 75]. Worldwide, about 38 million people are living with HIV infection at this time, with women making up 53% of the overall population affected [76]. HIV may be passed on to a baby before birth, during labor and delivery, or later in life via breast milk or other bodily fluids. HIV can also be passed on to adults through breast milk or other bodily fluids. As a result of this, HIV infection during pregnancy continues to be the leading cause of both the mortality of newborns as well as the lasting harm that it may cause [77]. Babies who are born to mothers who are infected with HIV have an exceptionally high chance of vertical transmission (up to 25% in the absence of antiretroviral medication). As a result, these infants are at risk for several major health issues such as AIDS and cardiovascular disease [78]. Nobody knows how HIV manages to get past the placental barrier. In addition, HIV infection is often accompanied by opportunistic infections, which might increase the likelihood of adverse results during pregnancy or vertical transmission of the virus [79].

Several studies have been conducted in recent years following significant advancements in highly active antiretroviral therapy (HAART). These studies have investigated potential differences between men and women regarding the progression of HIV infection, treatment response, and the pharmacokinetics of medications. Although there has been a slight decrease in HIV viral load among untreated women, particularly at elevated CD4 + levels, this difference does not warrant gender-specific recommendations for initiating therapy [80, 81]. Conversely, female participants exhibit a greater susceptibility to adverse events associated with antiretroviral therapy. Data on drug responsiveness indicates that treatment outcomes are comparable for both genders. However, there may be gender differences in treatment adherence and discontinuation for various reasons, including social and behavioral factors. According to the available evidence, women often experience greater pharmacokinetic exposure to antiretroviral medications than men [82, 83]. Key factors influencing this variance include P-glycoprotein activity, renal clearance, body weight, and body composition. Renal clearance is particularly significant. Numerous antiretroviral drugs affect P450 enzyme function, leading to potential drug interactions. Studies examining gender variations in the pharmacokinetics of anti-HIV drugs occasionally yield inconsistent results. Nevertheless, other mechanisms may account for the observed differences, complicating the inclusion of all potential confounding variables. When selecting anti-HIV regimens during pregnancy, it is crucial to carefully consider the various factors involved to protect HIV transmission and adverse outcomes for infants. Further research on gender differences in HIV medications is essential to maximize the effectiveness of therapy for all individuals infected with HIV. To achieve this goal, targeted research is necessary, and it is equally important to encourage the participation of women in clinical trials and cohort studies [84, 85].

### Zika virus and pregnancy

Global Zika activity has remained generally low since the 2015–2016 outbreaks. However, new outbreaks were reported in parts of Asia in 2024. In the Americas, over 3065 Zika cases had been reported by mid-March 2025, compared with 42,127 cases and two deaths in 2024. In the United States, 2025 has seen 4 travel-associated cases plus 1 case in a US territory, with no local mosquito-borne transmission. Meanwhile, Hawaii confirmed a travel-related Zika case (first since 2019), and authorities urge precautionary measures to prevent mosquito exposure—especially among pregnant individuals, due to risks like microcephaly [86].

The Zika virus (ZIKV) has been identified as spreading epidemically across regions including Africa, the Americas, Asia, and Europe. Typically, ZIKV is transmitted to individuals through mosquito bites [87, 88]. In pregnant adults, ZIKV infection may manifest with mild symptoms like low-grade fever, headaches, and rashes. However, this virus can cross the placenta, significantly increasing the risk of adverse pregnancy outcomes and developmental issues in newborns. These adverse outcomes encompass miscarriages, stillbirths, or the survival of infants with lifelong neurological abnormalities like enlarged ventricles, collapsing brains, or microcephaly [89].

**Fig. 2:**
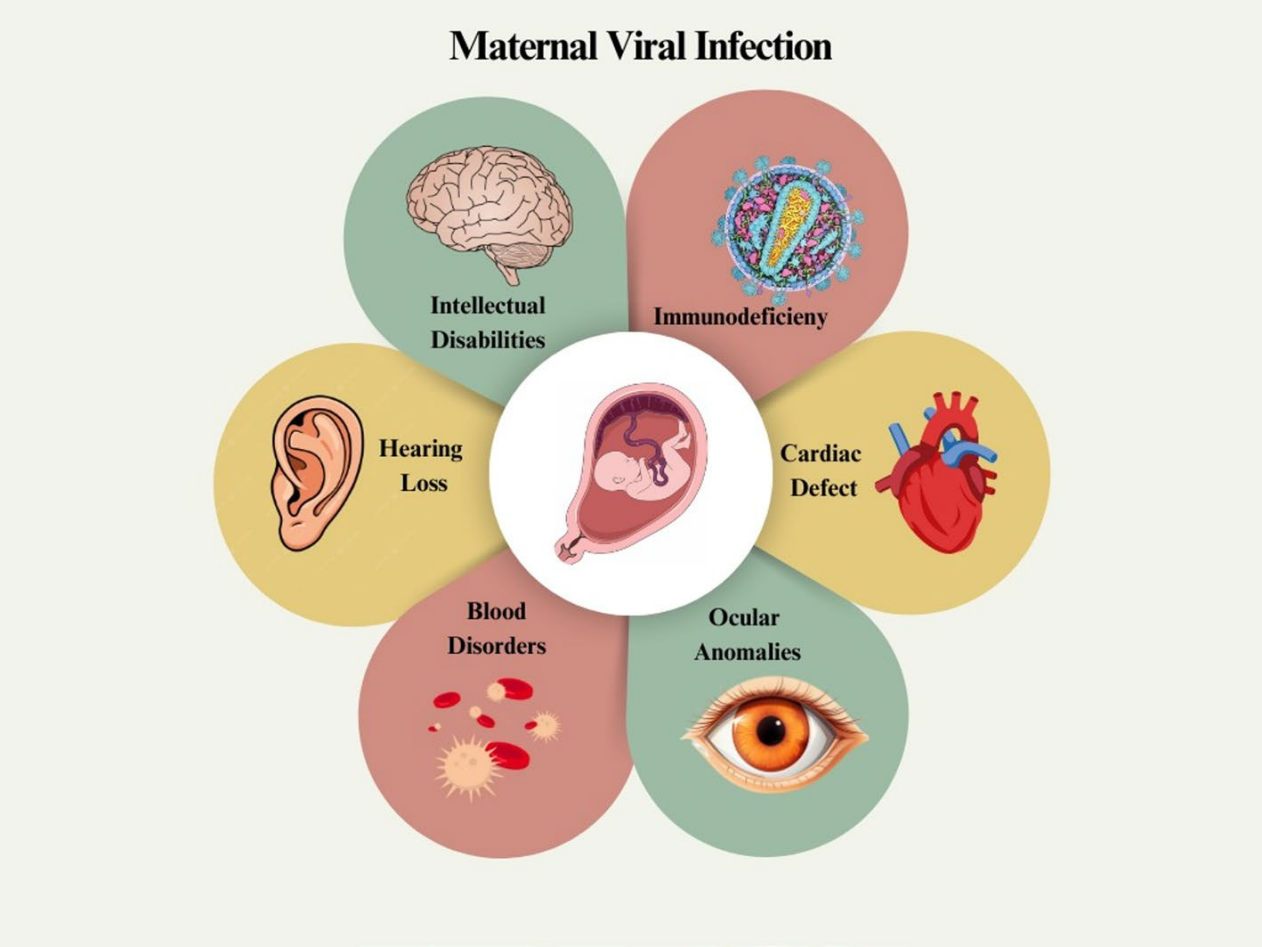
Represents the viruses that affect the fetus during pregnancy. Intellectual disabilities, blood disorders, immunodeficiency, and ocular anomalies are caused by ZIKV, CMV, RuV, and MSV

Researchers believe that ZIKV, which selectively infects various cells at the maternal-fetal interface and in the umbilical cord, and induces elevated levels of cytokines like IL-6, IL-15, IL-17, IFN-, CXCL10, and IFN- in amniotic fluid, could be responsible for severe fetal neurological abnormalities [90]. There is mounting evidence that ZIKV infection during pregnancy is linked to a birth defect known as congenital microcephaly. Babies born to mothers who contracted the Zika virus while pregnant are at risk of developing microcephaly [91]. This is because the stress on the endoplasmic reticulum in a developing brain can disrupt the normal unfolded protein response in the cerebral cortex [92]. The various types of viral infection are represented in Fig. 2.

### HPV and pregnancy outcomes

The prevalence of human papillomavirus (HPV) in pregnant women has been the subject of a significant number of research that have been carried out. When compared to women who are not pregnant, pregnant women have a greater chance of catching human papillomavirus (HPV), according to the results of these studies taken together. In their study, Liu and colleagues investigated the prevalence of human papillomavirus (HPV) in pregnant women and carried out an exhaustive assessment of the previous studies on the subject [93]. According to the findings of the study, 17.82% of pregnant women tested positive for HPV, whereas only 12.15% of women who were not pregnant tested positive. The study conducted by Luo and colleagues was conducted using a case-control methodology, and it comprised women of the same age who were either pregnant or not pregnant. According to the findings of this study, the prevalence of human papillomavirus (HPV) was significantly higher in the first group (24.2% vs. 14.8%) than it was in the second group [93]. Furthermore, it has been shown that HPV DNA can be detected in the placenta, amniotic fluid, and umbilical cord, suggesting a potential for vertical transmission to the newborn. This is noteworthy, considering that most studies on pregnant women have primarily focused on identifying infections in the uterine cervix. There is a consistent association between infectious diseases that occur during pregnancy and adverse pregnancy outcomes, as well as a strong correlation between these infections and significant repercussions for the infant [94]. Some studies indicate that there is no relationship between HPV and the progression and outcomes of pregnancy [95]. Conversely, other research has identified several adverse effects, including premature rupture of membranes [96], preterm birth, spontaneous abortion, pregnancy-induced hypertensive disorders [87], intrauterine growth restriction, low birth weight, and fetal death [97, 98]. The impact of human papillomavirus (HPV) on the course and outcomes of pregnancy remains unclear, as research has yielded conflicting results [99, 100].

Persistent infection with high-risk strains of human papillomavirus (HPV), particularly HPV-16 and HPV-18, is strongly associated with the integration of viral DNA into the host genome, a critical factor in the pathogenesis of cervical and other HPV-related cancers [101].

### Impact of SARS-CoV-2 infection during pregnancy

During the recent worldwide pandemic of COVID-19, over 308 million individuals were infected with the Severe Acute Respiratory Syndrome Coronavirus 2 (SARS-CoV-2) virus [102]. This infection led to the loss of more than 5.5 million lives. The SARS coronavirus type 2, often known as SARS-CoV-2, has the potential to cause severe harm to a wide variety of physiological and immune systems [103]. The health of pregnant women and the outcomes of their pregnancies are only two of the many physiological and immune systems that might be affected [104]. This virus may be passed from one person to another by intimate personal contact, most of the time, but it is also possible for it to be passed through aerosols and respiratory droplets. In the context of maternal and fetal tissues, the angiotensin-converting enzyme 2 (ACE2) receptor is present in a subpopulation of cells known as cytotrophoblast and syncytiotrophoblast [105]. This receptor is necessary for SARS-CoV-2 to connect to host cells and cause infection. The genetic material for the virus has been identified in placental and vaginal samples, but according to the most recent scientific findings, this does not yet represent proof of vertical transmission from mothers to their unborn kids [106]. The genetic material for the virus was found in placental and vaginal samples. This demonstrates that SARS-CoV-2 is unable to get through the placental barriers, even in instances when there is a severe infection in the mother [107]. Even though SARS-CoV-2 cannot be passed on to the developing foetus, it still has the potential to trigger an inflammatory reaction, which in turn might have a detrimental impact on the pregnancy and the growth of the baby [108]. After an infection, there is a rise in the levels of inflammatory cytokines in the mother’s blood. These cytokines include IL-1, IL-2, IL-7, IL-10, and TNF-. Maternal mortality, preeclampsia, and preterm delivery have all been associated to these higher levels. This connection may be traced back to the fact that both have a role in the body’s defensive mechanisms against infection and inflammation [109].

## Nanomedicine

Recent advancements in nanotechnology offer promising opportunities to develop innovative broad-spectrum nanotherapeutic platforms for addressing viral infections and overcoming associated challenges [110]. By loading antiviral drugs onto nanoparticles, we can enhance their bioavailability while reducing systemic toxicity. Moreover, targeted drug delivery holds the potential to further decrease toxicity, boost effectiveness, and extend the therapeutic window [111]. Some nanomaterials possess intrinsic toxicity that can effectively eliminate viruses. Recently, researchers have modified and engineered nanoparticles to adhere to pathogens and neutralize them selectively [112].

### HIV treatment through nanomedicines during pregnancy

There is now neither a vaccine nor a drug that can be used to fight HIV/AIDS. Neither option is currently available. Drugs are used to treat the illness by interfering with the virus at different stages of its life cycle. This allows the virus to no longer replicate and spread. Because of this, the virus is unable to reproduce and will no longer propagate [113]. There are now six different antiretroviral medicines available on the market. ARV is an abbreviation that refers to antiretroviral drugs [110]. Several examples include the following: Antiviral medications include nucleoside/nucleotide reverse transcriptase inhibitors (also known as N(t) RTIs), integrase inhibitors, CCR5 antagonists, protease inhibitors, entry/fusion inhibitors, and protease inhibitors. If one so chooses, they are able to get their hands on any one of these drugs. The highly active antiretroviral therapy, more often referred to as HAART, is a treatment for HIV/AIDS that consists of a combination of at least three different antiretroviral medicines. Since it was first used, HAART has resulted in considerable improvements not just to patients’ life expectancy but also to their overall quality of life [114]. The Human Assisted Animal Rescue Team (HAART) was founded sometime around the 1990s. The fact that there are drawbacks to the medications that are already available, such as poor adherence, enormous daily doses, toxicity, and other unpleasant side effects, does not cancel out the advantages of the therapies that are currently being provided [115]. Because HIV infection is a chronic condition, it is also possible for patients to acquire drug resistance if they are required to take medication for the rest of their lives. This is because HIV infection is a chronic condition [115]. This is due to the fact that HIV infection and AIDS are both chronic illnesses. As a direct consequence of this, it is of the highest importance to do research on and come up with original approaches to the prevention of HIV [116].

Accelerating research on nanotechnology-based methods of administering medications is of the highest relevance for the treatment of HIV in pregnancy [117]. The modern design of drugs has the potential to enhance the ARV treatment in terms of both its safety and its efficacy if nanosystems are included into the process of administering the medicine [118]. It is conceivable to minimise the dosage needs of the present large pill loads, as well as their negative side effects (and, as a consequence, the likelihood of drug resistance) [119]. This will allow the aim to be achieved.

Chiodo et al. [120] performed research to explore the in vitro efficacy of the NRTI drugs abacavir (ABC) and lamivudine (3TC) when they were paired to glucose-coated GNPs. The findings of this study showed that both medications were equally effective. The major hydroxyl groups in the drugs were ingeniously functionalized in such a way as to generate an ester link. This was done to prevent viral replication in acidic environments, such as the vagina. This action was taken to ensure that the drugs would not be rendered useless. This was done to promote chain termination, which is a crucial stage in the action mechanism of the NRTI drug family. To achieve this objective, the main hydroxyl groups of the medicines were intentionally functionalized. These findings suggest that GNPs have the potential to be useful as multivalent pharmaceutical delivery strategies in the treatment and care of HIV patients.

Treg cells, also known as regulatory T cells, are a subtype of T cells that are vital to the process of keeping the immune system in check and functioning properly [121]. Despite this, these cells are vulnerable to HIV infection due to the unique properties that they contain, which make them stand out from other types of cells. The immunological hyperactivation that is brought on by HIV can reduce the amount of T-cells in a person’s body, in addition to causing feelings of fatigue and sleepiness. T regulatory cells, sometimes referred to as Treg cells, are of the highest relevance in the setting of HIV infection due to their ability to reduce both excessive immunological activation and inflammation [122]. This makes T regulatory cells one of the most important immune cell subsets. The study that was carried out by Jaramillo-Ruiz and colleagues is regarded as being groundbreaking since it demonstrates the power of carbosilane dendrimers to protect Treg cells against HIV infection in vitro [123]. By administering these dendrimers, we were able to mitigate the HIV infection-induced impairments in both the phenotypic and functional characteristics of these cells, and we were successful in doing so. Studies that were carried out in vitro and in vivo demonstrated a considerable decrease in the formation of p24 antigen, which is a clear indication of an extremely high level of biocompatibility [124].

By affixing RNA decoys to dendrimers, the researchers from Parboosing and their colleagues hoped to interfere with the life cycle of the HIV virus [125]. The third stem loop of the HIV packaging signal was utilized in the manufacture of these RNA decoys, which resulted in the development of a 16-mer oligoribonucleotide. RNA decoys are used to fool the body into thinking that a virus is present when it is not. According to these data, it would seem that cytoprotecting had only a little effect on the HIV infection and that intracellular transport was accomplished without any problems [126].

An antiretroviral drug known as tenofovir and a prospective therapy for reversing the effects of latency known as vorinostat may be co-encapsulated in ultrasmall iron oxide nanoparticles (10 3 nm), according to the study that Jayant and his colleagues carried out [127]. During this study, latent HIV was reactivated in human astrocyte cultures. Additionally, the amount of time it took for drugs to reach circulation was lengthened by 30% for five days [128]. Additional studies showed that there was an increase in both BBB transmigration and antiviral activity in vitro [129]. There has been a considerable amount of research conducted on nanoparticles as prospective innovative agents for the delivery of antiretroviral medications, other small-molecule HIV inhibitors, and vaccines [130].

### Hepatitis B virus (HBV) and nanomedicine

Hepatitis B (HBV): Approximately 254 million people were living with chronic HBV as of 2022, with an additional 1.2 million new acute cases that year. Prevalence varies significantly by region, reaching around 7.5% in Africa and as low as 0.5% in the Americas. Hepatitis C (HCV): Roughly 130–180 million people worldwide, a bit over 3% of the global population, are affected by chronic HCV infections. Hepatitis E (HEV). The most recent figures (2021) estimate around 19.5 million acute HEV infections globally and about 3450 deaths. HEV accounts for 5.4% of global acute hepatitis DALYs [131]. High-risk areas include sub-Saharan Africa and parts of South and East Asia. Notably, HEV infection during pregnancy can yield maternal fatality rates up to 20–25% in the third trimester. Certain countries, such as China and Pakistan, have licensed the HEV 239 (Hecolin) vaccine, although global WHO prequalification and broader rollout, particularly among pregnant women and immunocompromised groups, are still pending [132]. Individuals throughout the globe have chronic infections due to HBV, a virus that may cause liver inflammation. It may lead to serious health problems, including cirrhosis and liver cancer, which kill about 780,000 people annually [129]. There are several limitations to the existing HBV treatments, which include interferon, pegylated interferon, lamivudine, adefovir, entecavir, telbivudine, and tenofovir. High costs, severe side effects, the risk of liver failure during flare-ups, and medication resistance are only some of the downsides. Potential new therapies for HBV are being studied using nanotechnology [133]. Wang and his colleagues conducted several trials with various cationic nanoparticles made from biodegradable polymers as part of their study [134]. Two techniques were employed to create these nanoparticles: nanoprecipitation and solvent evaporation. To diminish hepatitis B surface antigen (HBsAg) production, this research aimed to assess how effectively they could carry siRNA and DNA [135]. Methoxy poly (ethylene glycol)-poly(lactide) (mPEG-PLA) nanoparticles with a polyethyleneimine [39] coating were shown to be the most effective against HBV. It was shown that the efficiency of siRNA delivery was affected by both the size and surface charge of the nanoparticles [135].

### Hepatitis C virus

**Fig. 3:**
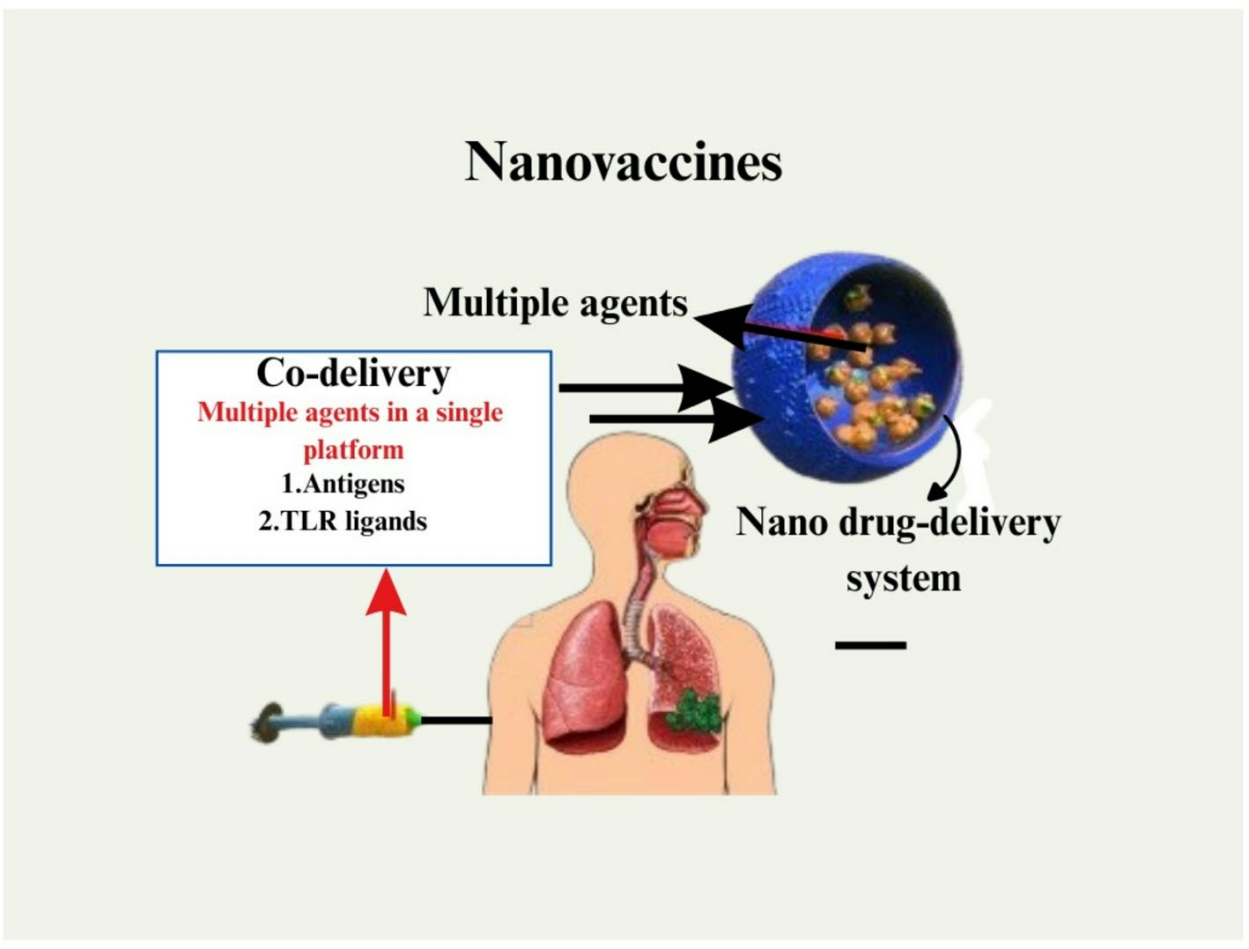
Represent the nanoparticle in the form of a nano vaccine against the viral infection by promoting the immune response. Nano vaccines enable co-delivery of antigens and TLR ligands using nano drug-delivery systems to improve immune targeting

It is estimated that between 130 and 150 million people throughout the world are infected with the hepatitis C virus (HCV), which may lead to cirrhosis of the liver or cancer of the liver [136]. Every year, HCV-related liver disease claims the lives of around half a million people in the United States. For nano-level HCV treatment, the use of PEGylated interferon (PEG-IFN) in conjunction with ribavirin is considered the gold standard [137]. Peginterferon alpha-2a (Pegasys^®^) was granted approval by the Food and Drug Administration (FDA) as a therapy for HCV in the year 2002. Peginterferon alpha-2b (PegIntron^®^) had already been commercially available since the year 2001. PegIntron^®^, which has a molecular mass of 31 kDa, has been shown to have superior clinical outcomes than the smaller, un-PEGylated variant of IFN-2b (19 kDa). This is because PegIntron’s molecular mass is due to its PEGylation process [138]. For the treatment of HCV, researchers have been looking at new nanotechnology-based medicinal approaches. They were able to demonstrate that the successful coupling of IFN to gold nanoparticles (GNPs) could be achieved by physical binding and complexation with hyaluronic acid (HA). This resulted in long-acting nano-complexes that could be administered in animals [139]. Figure 3 represents the nano-vaccines and their effect.

models for targeted treatment. As a result of the fact that these nano-complexes stayed in the liver for up to 7 days after injection, there is reason to believe that HCV treatment might be enhanced and extended over time. Over 99% of HCV was suppressed, according to the results of Wang et al. [140] who produced nanozymes from GNPs functionalized with RNAse A and anti-HCV oligonucleotides. These nanozymes were able to withstand treatment with proteinase, were effectively internalized, and shown a low level of toxicity. Another set of researchers employed something called cross-linked polymeric micelles (CLPM) to target the HCV that was contained inside test tubes [141]. Micelles were used to encapsulate camptothecin (CPT), a potent anti-HCV molecule, to circumvent the molecule’s deficiencies, which include poor water solubility and chemical instability. CLPMs formed amphiphilic micelles with a hydrophobic core and a hydrophilic shell to maintain the efficiency of the HCV antiviral treatment while also reducing the cytotoxicity [142]. The researchers Moon et al. [143] looked into the possibility of using siRNA that was coupled to lipidoid nanoparticles in order to target proviral host components that are necessary for HCV replication. Patients who do not reply to typical HCV drug regimens may have reason for optimism owing to the powerful anti-HCV effect revealed by these lipid-like delivery molecules over the course of several days in mouse models [144]. Because of their relatively low toxicity, cationic liposomes, and cholesterol-based liposomes in particular, are excellent candidates for application in clinical settings. In order to direct the delivery of inhibitory siRNA to the liver, cholesterol-based cationic liposomes were used [145]. These liposomes included the lipid-soluble antioxidant form of vitamin E (-tocopherol). This approach was successful in lowering levels of HCV core antigen as well as the activity of the reporter gene firefly luciferase, which is used to determine the extent to which HCV replication has occurred [146].

### Herpes simplex virus (HSV) and nanomedicine

Common ailments caused by herpes simplex virus (HSV) include orofacial lesions, encephalitis (usually due to HSV-1), vaginal infections (often due to HSV-2), and disseminated disease [147]. Treatment for herpes simplex virus (HSV) infections often involves the use of acyclovir. Precursor medications like valacyclovir and famciclovir are known for their improved bioavailability [148]. Acyclovir may be taken orally, intravenously, or used topically; however, there are limitations to each route of administration [149]. Examples include the low bioavailability (15–30%) of oral acyclovir treatment, patient compliance issues, and the limited skin permeability of topical acyclovir use [150]. To boost the effectiveness and absorption of poorly absorbed medications like acyclovir, researchers have explored the use of buccal drug delivery as an alternative method. This action was taken in response to the aforementioned concerns [151]. Researchers investigated the efficacy of acyclovir delivered by buccal administration utilizing nanospheres as delivery agents. In vivo studies on rabbits have demonstrated that the drug absorption rate may be greatly enhanced by administering acyclovir-loaded nanospheres orally as opposed to the free drug [152]. The maximum drug concentration was increased from two hours to six hours, and the peak plasma concentrations were three times higher than previously, suggesting a potential decrease in dosing frequency [153]. Multiple studies have shown not only the inherent antiviral properties of silver nanoparticles, but also how loading nanoparticles with acyclovir may boost the degree of HSV suppression that can be accomplished [116]. Additionally, it has been shown that anti-herpetic siRNA-loaded nanoparticles increase bioavailability in mice, and that acyclovir-loaded nanoparticles increase permeability across vaginal membranes in rat models, leading to greater tissue distribution and bioavailability compared to the medication in its free form [154]. This new knowledge has substantial bearing on the therapeutic management of HSV, especially among women. Consistent with these findings, further in vivo studies have showed prolonged drug retention times and improved skin dispersion [155].

### Human cytomegalovirus treatment through nanoparticles

Cytomegalovirus (CMV), a herpesvirus, poses significant health risks, particularly in neonates and those with compromised immune systems [156]. Currently, there is no vaccine available to prevent CMV infection, and the existing antiviral drugs are plagued by issues such as toxicity, limited effectiveness, and susceptibility to drug resistance [157]. In this study, researchers conducted a chemical conjugation between a monoclonal antibody designed to target CMV’s surface glycoprotein [119] and gold nanoparticles (GNP) [158]. They investigated the potential of this gB-GNP conjugate to act as an antiviral agent against CMV [159]. The findings revealed that the gB-GNP conjugate effectively hinders CMV replication, prevents virus-induced damage to cells, and curbs the spread of the virus in cell cultures, all without causing harm to the host cells.The antiviral activity of the gB-GNP conjugate was concentration-dependent [160]. At higher concentrations, it formed a protective coating on the surface of virus particles, preventing them from entering host cells. In contrast, at lower concentrations, it disrupted a later stage of the virus’s life cycle. In summary, this research suggests that the gB-GNP conjugate holds promise as a potential antiviral approach against CMV infection [161].

### Human papillomavirus

Epithelial cell manifestations of human papillomavirus (HPV) infections range from warts to cervical neoplasia and carcinoma [162]. There are over a hundred distinct types of HPV, albeit only a small percentage of them are thought to be harmful. It is believed that HPV16 and HPV18, two high-risk genotypes, are responsible for 70% of cervical cancer cases, making Cervisil^®^ (STP909) a promising nanobased treatment option [163]. For both HPV16 and HPV18, STP909 has been shown in in vitro studies to form robust duplexes with the E7 mRNA. In vivo studies in rabbits have revealed that these nanoparticles can effectively target and block the E7 gene utilising knock-down technology [164]. Not only has HPV caused anxiety among scientists, but so have other viral outbreaks, such Ebola in 2013 and Zika in 2015. To address these demands, several nanoformulations have been developed. Since PLGA polymeric nanoparticles are biocompatible and biodegradable, they were packed with the oral drug ivermectin by the research team. The Zika virus was the target of these nanoparticles’ antiviral properties [165]. To prevent damage from stomach acid, PEG was included into the nanoparticles’ outer layer. The nanoparticles’ incorporation of the antibodies’ Fc regions facilitated their passage through the epithelial barrier and into the bloodstream [166]. When these nanoparticles were administered orally to mice, over 65% were able to enter the circulation through Fc-mediated transcytosis. The Zika virus’ nonstructural protein 1 was considerably suppressed by these nanoparticles in the lab, and the formulation was acid-stable (pH 3) and lyophilizable for capsule packing [167].

### Nanoparticles in SARS-CoV-2 infection and women health

The distribution efficiency of repurposed antiviral drugs may be improved by incorporating cell-penetrating peptides (CPPs) into nanocarriers [168]. Among the several endocytosis mechanisms, clathrin-protein-poor (CPP) molecules may facilitate micropinocytosis, caveolae-mediated endocytosis, and clathrin-independent endocytosis. The effectiveness and specificity of cargo delivery may be improved by combining these CPPs with multifunctional polymeric nanoparticles (NPs) or lipid nanoparticles (LNPs) to protect the payload from proteases [169]. This method is now being studied as a potential therapy for SARS-CoV-2 infection. Conjugating CPPs to Tat peptides and delivering them via nano formulation-based NP delivery methods is an area of active research. Enzymatic breakdown, rapid clearance, and trouble crossing the cell membrane are only some of the issues plaguing current small interfering RNA (siRNA) therapies [170]. One strategy to effectively address these challenges is to make use of a broad range of NPs, including but not limited to LNPs, polymeric NPs, hybrid NPs, nanohydrogels, superparamagnetic iron-oxide NPs (SPIONs), and functionalized gold nanoparticles (AuNPs) [171]. In vivo siRNA delivery is facilitated by the widespread usage of polymers such as polyglutamic acid (PGA), poly(lactic acid) (PLA), polycaprolactone (PCL), and its copolymers like PLGA. The Food and Drug Administration has reviewed and approved these polymers for use [172]. The most efficient method of administering an aerosolized siRNA NP delivery system using a metered-dose inhaler is recommended for the treatment of SARS-CoV-2 [173]. It has also been looked into whether or not silica/polyP NPs can be employed to protect polyP from the degradation brought on by alkaline phosphatase [174]. The goal of this approach is to inhibit the interaction between the S protein of SARS-CoV-2 and the ACE2 receptor, with the hope that this would enhance the innate immune response of humans infected with the virus [175]. Long-acting DNase-1 coated PDA-poly (ethylene glycol) NPs were developed to decrease cfDNA and enhance neutrophil activities in different lines of study. These NPs provide hope for a therapeutic solution to the problem of COVID-19 [176]. These NPs have the potential to halt the progression of sepsis in COVID-19 patients due to their capacity to inhibit cfDNA [177]. Chitosan nanoparticles (NPs) designed for inhalational delivery have also been created [178]. This permits targeted delivery of the medicine to lung epithelial tissues in conjunction with controlled release, lowering the drug’s toxicity. Novochizol is a kind of nanoparticle (NP) chitosan designed to encapsulate a wide range of drugs for the treatment of individuals with acute COVID-19 [179]. Antiviral drugs like hydroxychloroquine (HCQ) and chloroquine [104] have been studied in combination with nanoparticles of silver (AgNPs), gold (AuNPs), silver-gold (AgAu-NPs), and platinum (Pt NPs) to enhance targeted therapy and effectiveness in COVID-19 treatment while minimising side effects [180, 181].

#### Short coming of nanoparticles in maternal viral infection

NPs as delivery systems have proved to have considerable potential for enhancing the prevention and treatment of maternal viral infections; nevertheless, various challenges continue to prevent their clinical translation. Long-term safety of these nanoparticles is one of the key issues because, in some cases, they may cause unpredictable toxicity, immunogenicity, or tissue accumulation of nanoparticles at other sites. The importance of this problem is even more pressing in the case of pregnancy, as the well-being of a mother and a fetus should be ensured. Moreover, most of the formulations of the nanoparticles exist in biological fluids in an unstable state, which causes the early release or degradation of the therapeutic load of nanoparticles at an incorrect location [182].

The other shortcoming rests on the complexity of the maternal-fetal interface. The placental barrier is protective, but at the same time, it presents a significant obstacle to effective and selective delivery. The optimal targeting scheme to achieve the optimal maternal-to-fetal balance has not been perfected yet in existing nanoparticles. Moreover, the pharmacokinetics and biodistribution of nanoparticles during pregnancy are still not well-defined, and it is challenging to make projections regarding their behavior, effectiveness, and toxicity in vivo [183].

From a future perspective, a number of techniques can be used in order to counteract these challenges. Nanoparticle engineering innovations, which may include surface functionalization with biocompatible polymers or ligand conjugation and generation of stimuli-responsive nanocarriers, would have the potential to increase precision and safety. Using Stein during the production of fabrics may also eliminate the risks of toxicity. Furthermore, the synergistic combination of nanoparticles with state-of-the-art modes of action, either mRNA therapeutics or CRISPR-based antivirals, or immune-modulating compounds, may provide novel means to more effective maternal-fetal interventions [184].

Standardized frameworks to assess the safety and efficacy of nanoparticles during pregnancy also need to be adopted to give them greater clinical utility. This encompasses thorough preclinical research in the appropriate models, close examination of the results on maternal and fetal outcomes, and alignment of regulation on translational research. Therefore, research into nanoparticle-based delivery systems has the potential to be used as a potent solution to curb and prevent maternal viral infections in the future, given that the limitations are addressed and the aspect of innovation is put forth [185].

#### Challenges with nanoparticles with maternal viral infection

Nanoparticle-based delivery systems have shown remarkable potential for improving the prevention and treatment of maternal viral infections; however, several limitations still hinder their clinical translation. One of the major challenges is their long-term safety profile, as nanoparticles can sometimes trigger unexpected toxicity, immunogenicity, or accumulation in non-target tissues. In the context of pregnancy, this issue becomes even more critical, since both maternal and fetal safety must be ensured. Additionally, many nanoparticle formulations face stability challenges in biological fluids, leading to premature release or degradation of the therapeutic payload before reaching the intended site of action [186].

Another limitation lies in the complexity of the maternal–fetal interface. The placental barrier, while protective, poses a major challenge to efficient and selective drug delivery. Achieving the right balance between maternal treatment and fetal safety requires precise targeting strategies, which current nanoparticles have not fully optimized. Furthermore, the pharmacokinetics and biodistribution of nanoparticles in pregnant individuals remain poorly characterized, making it difficult to predict their behavior, efficacy, and safety in real-world scenarios [187].

Looking toward the future, several strategies may help overcome these challenges. Advances in nanoparticle engineering, such as surface functionalization with biocompatible polymers, ligand conjugation for receptor-mediated targeting, and the development of stimuli-responsive nanocarriers, could enhance precision and safety. Incorporating biodegradable and naturally derived materials may further reduce toxicity risks. Moreover, combining nanoparticles with cutting-edge modalities such as mRNA therapeutics, CRISPR-based antivirals, or immune-modulating agents could open new avenues for more effective maternal–fetal interventions [188].

To strengthen their clinical utility, standardized frameworks for assessing nanoparticle safety and efficacy during pregnancy must also be established. This includes comprehensive preclinical studies in relevant models, careful evaluation of maternal and fetal outcomes, and harmonized regulatory guidelines for translational research. By addressing these limitations and focusing on innovation, nanoparticle-based delivery systems can evolve into powerful tools for mitigating and preventing maternal viral infections in the future [189].

## Conclusion

When a mother gets infected while she is carrying her child, there is an increased risk that the child may have long-term health problems such as autism spectrum disorder (ASD), schizophrenia, social difficulties, cognitive impairment, and perhaps even other illnesses. The author of this essay examines two of the most significant ways in which the illness of a woman might impede the mental development of her kid. The so-called “passive effect” is an example of one such strategy. First, the virus is able to infect cells that are necessary for neurogenesis when it is passed vertically via the placenta. This process occurs throughout pregnancy. Microglia and neuron-like progenitor cells (NPCs) are two examples of such cells. Second, if a woman has a viral infection, her systemic circulation is flooded with pro-inflammatory cytokines, which may make the condition much worse. It is well established that these cytokines make their way into the circulatory system of the growing fetus as well as the brain. There is reason to believe that NPs will pave the way for the creation of brand-new methods in addition to the enhancement of existing treatments. Because of the innovative methods of action that these novel modalities use, it is possible that they will be able to destroy viruses or halt the spread of viruses. Because of their one-of-a-kind characteristics, nanoparticles (NPs) provide a broad variety of benefits; these benefits, in turn, might be leveraged to increase the potency of antiviral medications. NPs are able to preserve their payloads from systemic degradation and minimize cytotoxicity because their payloads are less exposed to the outside environment than those carried by free particles. In addition, nanoparticles may be able to prevent the spread of the virus by passing through the placenta and into the circulation of the fetus. In the case that either the mother or the kid becomes unwell, this is one of the most effective strategies to protect both from any potential danger.

## Data Availability

No datasets were generated or analysed during the current study.
